# A Particle Swarm Optimization Variant with an Inner Variable Learning Strategy

**DOI:** 10.1155/2014/713490

**Published:** 2014-01-23

**Authors:** Guohua Wu, Witold Pedrycz, Manhao Ma, Dishan Qiu, Haifeng Li, Jin Liu

**Affiliations:** ^1^Science and Technology on Information Systems Engineering Laboratory, National University of Defense Technology, Changsha, Hunan 410073, China; ^2^Department of Electrical & Computer Engineering, University of Alberta, Edmonton, AB, Canada T6R 2V4; ^3^Warsaw School of Information Technology, Newelska, 01-447 Warsaw, Poland; ^4^Department of Electrical and Computer Engineering, Faculty of Engineering, King Abdulaziz University, Jeddah 21589, Saudi Arabia; ^5^School of Civil Engineering and Architecture, Central South University, Changsha, Hunan 410004, China

## Abstract

Although Particle Swarm Optimization (PSO) has demonstrated competitive performance in solving global optimization problems, it exhibits some limitations when dealing with optimization problems with high dimensionality and complex landscape. In this paper, we integrate some problem-oriented knowledge into the design of a certain PSO variant. The resulting novel PSO algorithm with an inner variable learning strategy (PSO-IVL) is particularly efficient for optimizing functions with symmetric variables. Symmetric variables of the optimized function have to satisfy a certain quantitative relation. Based on this knowledge, the inner variable learning (IVL) strategy helps the particle to inspect the relation among its inner variables, determine the exemplar variable for all other variables, and then make each variable learn from the exemplar variable in terms of their quantitative relations. In addition, we design a new trap detection and jumping out strategy to help particles escape from local optima. The trap detection operation is employed at the level of individual particles whereas the trap jumping out strategy is adaptive in its nature. Experimental simulations completed for some representative optimization functions demonstrate the excellent performance of PSO-IVL. The effectiveness of the PSO-IVL stresses a usefulness of augmenting evolutionary algorithms by problem-oriented domain knowledge.

## 1. Introduction

Optimization plays an important role in scientific research, management, industry, and so forth, given the fact that many problems in the real world are essentially optimization tasks. However, with the increase of complexity of optimization problems associated with multimodality, noise, and high dimensionality of problems, “traditional” optimization methods (e.g., gradient-based methods) are no longer completely effective when searching for optimal or satisfactory solutions within the bounds of reasonable computation cost. In light of these challenges, many bioinspired algorithms, such as Genetic Algorithms (GAs) and Ant Colony Optimization (ACO), have emerged. Particle Swarm Optimization (PSO), developed by Kennedy and Eberhart [1, 2], is a competitive population-based algorithm being particularly efficient when dealing with continuous optimization problems. It is a swarm intelligence [3] algorithm that emulates swarm behaviors such as birds flocking and fish schooling [4]. Each particle in PSO adjusts its flying speed and direction by learning from its own past experience and neighbors' experience, attempting to search for better position gradually [5].

Due to its powerful capability and relatively low number of parameters, PSO has drawn wide attention since its inception. To enhance the efficiency of the generic version of the PSO method, many variants have been presented. These variants are realized through different augmentations of the generic method, generally including parameter tuning [6–11], topology structure adjustment [12–16], intelligent combination of various search strategies [17–19], and hybridization with other classical optimization techniques [20–23]. Although significant progress and achievements have been obtained, still a fundamental challenge on how to make PSO successful in determining optimal or near optimal solutions for optimization problems with complicated landscapes and of high dimensionality still remains. In addition, even though PSO has been praised for many merits, including simple implementation, it has been criticized for suffering from premature and the quick performance degradation in case of increasing dimensionality of the optimization problem [24].

Noticeably, previous PSO variants generally focus on the modification of particle's behaviors, to strengthen simultaneously its exploration and exploitation capabilities. These efforts indeed improve significantly the effectiveness of the generic PSO. Another promising direction in improving the PSO performance is to acquire and utilize the domain knowledge associated with the optimization problems at hand. Subsequently this domain knowledge can be integrated into the search strategy in anticipation of delivering more effective search guidance for the particles. As a matter of fact, the combination of knowledge-based strategy with the heuristics of swarm optimization has been demonstrated to be effective in discrete optimization [25–27]. Note that the problem domain knowledge in discrete optimization (e.g., the scheduling problem [26, 27] and the spatial geoinformation services composition problem [28, 29]) is dependent on concrete problems considered and the knowledge extraction and discovery process is relatively subjective. In [30], the authors proposed a variable reduction strategy by utilizing the knowledge of derivative equations of unconstraint optimization problems, to reduce the complexity of original optimization problems.

The notion of variable symmetry can be encountered in optimization functions. Variable symmetry means that all or some variables encountered in the function under optimization are symmetric; namely, they can exchange positions through linear transformation without affecting the original function. We refer to such functions in which all variables are symmetric as completely symmetric function. There are functions in which only some variables are symmetric giving rise to the concept of partially symmetric function. In general, symmetric functions are developed by using operators of summation and product (“∑” and “∏”). According to this observation, we note that all symmetric variables in the optimal solutions of such a function are supposed to satisfy a certain quantitative relation. The domain knowledge acquired about symmetric functions becomes useful in the enhancement of the search performance. The underlying motivation of this study is to utilize such domain knowledge to strengthen the PSO's capability in solving optimization problems with symmetric variables.

The major contributions of the paper can be summarized as follows.

1. Based on the knowledge that symmetric variables in the optimal solution of an optimization function satisfy a certain quantitative relation, we present an inner variable learning (IVL) strategy to provide particles with exact and efficient search guidance.
2. We design a trap detection strategy, by which one can determine if the particle has been trapped in a local optimum. We also employ an adaptive Gaussian mutation-based trap jumping out strategy to help particles to escape from local optima.
3. We propose a new knowledge-driven PSO variant, named PSO-IVL, which is integrated with the IVL strategy, trap detection and jumping out strategy, and the basic PSO.
4. Extensive experimental simulations and analysis are conducted to demonstrate the efficiency of PSO-IVL in solving global optimization functions with symmetric variables and offer a comparison with some other state-of-the-art PSO variants.

The paper is structured as follows.  Section 2 briefly introduces the basic PSO and reviews related work existing in the literature.  Section 3 details the IVL strategy.  Section 4 introduces the trap detection and jumping out strategy and proposes the algorithm framework of PSO-IVL.  Section 5 reports experimental simulations and offers a detailed performance analysis.  Section 6 concludes this paper identifying future research directions.

## 2. Related Studies

PSO has undergone significant progress since its introduction in 1995. A large number of PSO variants have been proposed to improve the performance of traditional PSO. Comprehensive reviews of PSO can be found in [36–38]. In addition, Valle et al. [39] surveyed PSO along with its basic concepts, variants, and applications in power systems. Rana et al. [40] reviewed PSO and its application to data clustering. In this section, we first briefly introduce the basic PSO and then survey the major PSO variants.

### 2.1. Basic PSO

Analogous to some other evolutionary algorithms, such as Genetic Algorithm and Ant Colony Optimization, PSO is a population-based stochastic optimization algorithm. A swarm of particles in PSO attempt to search for superior solutions through learning, communication, and interaction. The position of each particle refers to a solution. Then the position moving process of a particle in the solution space relates to a solution search process. The state of particle *i* is described by its current position **x**_**i**_ = [*x*_*i*1_, *x*_*i*2_,…, *x*_*iD*_] and velocity **v**_**i**_ = [*v*_*i*1_, *v*_*i*2_,…, *v*_*iD*_], where *D* stands for the number of variables encountered in the optimization problem. In the generic PSO with inertia weight [31], the position and velocity of particle *i* are updated during the evolutionary process:

(1) vid′⟵w×vid+c1×r1d×(pBestid−xid)   +c2×r2d×(gBestd−xid),

(2) $$\begin{array}{c} x_{id}^{\prime }⟵x_{id}+v_{id}^{\prime }, \end{array}$$

where *x*_*id*_′ and *x*_*id*_ represent the *d*th variable (or dimension) of the next and current positions of particle *i*; *v*_*id*_′ and *v*_*id*_ denote the *d*th variable of the next and current velocities of particle *i*; *p*Best_*id*_ is the *d*th variable of the personal historical best position found by particle *i* up to now, and *g*Best_*d*_ is the *d*th variable of the global best position found by the overall particles so far; *c*_1_ and *c*_2_ are acceleration parameters which are commonly set to 2.0; *r*_1*d*_ and *r*_2*d*_ are two random numbers drawn from a uniform distribution over [0,1]; and *w* denotes the inertia weight, which is used to set up the balance between the abilities of global and local search features of PSO. The inertia weight parameter is widely adopted by major PSO variants [31].

The behavior of the particle is specified by its velocity and position update realized according to (1) and (2) [39, 41]. The first inertia weight component of (1) models the tendency of the particle to continue in the same direction as before. The second component of (1) is referred to as the particle's “memory,” “self-knowledge,” “nostalgia,” or “remembrance” [39, 41]. It reflects the self-learning behavior of the particle. The third component in (1) is referred to as “cooperation,” “social knowledge,” “group knowledge,” or “shared information” [39, 41]. It reflects the social learning behavior of the particle. Equation (2) indicates that the position of the particle in the solution space will be changed in terms of its current position and next velocity.

After each update, we check the position and velocity of each particle to guarantee them being within a predefined certain range. In our study, if the position and velocity exceed the range, they are modified as follows:

(3) $$\begin{array}{c} v_{id}^{\prime }⟵\min\left(v_{d}^{\max},\max\left(v_{d}^{\min},v_{id}^{\prime }\right)\right),\\ x_{id}^{\prime }⟵\bar{p\text{Bes}{\text{t}}_{id}}, \end{array}$$

where *v*_*d*_^max⁡^ and *v*_*d*_^min⁡^ are maximum and minimum value of *d*th variable of the velocities, respectively. $\bar{p\text{Bes}{\text{t}}_{id}}$ is the mean value of *d*th variable of the personal historical best positions of all particles.

### 2.2. Major PSO Variants

“Standard” PSO exhibits some deficiencies, including suffering from being premature and inefficient in solving complex multimodal optimization problems. One way to strengthen the capability of PSO is to dynamically adapt its parameters when running the particles' evolutionary process. The inertia weight parameter *w* is set to linearly decrease over iterations [31, 42]. In addition, a fuzzy adaptive mechanism was used to tune the value of *w* [9]. Kennedy and Eberhart recommended that the proper value for the acceleration parameters *c*_1_ and *c*_2_ could be fixed and set to 2.0. These values were adopted in many works. In comparison, Suganthan [13] suggested that the usage of ad hoc selected values for *c*_1_ and *c*_2_ rather than the fixed value for different problems could result in better performance. Ratnaweera et al. [8] presented a PSO variant with linearly time-varying acceleration coefficients (HPSO-TVAC). Zhan et al. [17] proposed an adaptive PSO, which enables the automatic control of inertia weight, acceleration coefficients, and other algorithmic parameters at run time according to four evolutionary states, that is, exploration, exploitation, convergence, and jumping out state. Ismail and Engelbrecht [10] controlled the parameters of PSO by embedding them in the position vector of particles, which enhanced the performance of comprehensive learning PSO (CLPSO) [35].

Besides parameter adaptation, topological structures of the particle swarm were also extensively studied. For example, Kennedy [12, 16] suggested that a small neighborhood might be more suitable to complicated multimodal problems while a larger neighborhood might be more effective for relatively simple unimodal problems. In [16], Kennedy and Mendes evaluated some typical topologies including global best topology, ring topology, wheel topology, pyramid topology, and Von Neumann topology. They suggested that the Von Neumann topology configuration may perform better compared to others. However, the selection of an appropriate neighborhood structure is generally problem oriented. Being aware of the noticeable effect of neighborhood structures, the neighborhood structure dynamic adaptation mechanisms were also investigated by some researchers [13, 43]. Mendes et al. [33] presented a fully informed particle swarm (FIPS) in which each individual learns the experience of all its neighbors rather than just the best one and itself.

Another natural evolution of the Particle Swarm Optimization can be achieved by incorporating operators or techniques that are effectively used in other evolutionary algorithms [39]. Angeline [20] developed a hybrid PSO by introducing the selection operator coming from Genetic Algorithm. A hybrid PSO based on genetic programming was presented by Poli et al. [23]. In [44], Juang integrated GA with PSO for designing artificial neural network. Other operators and techniques, such as crossover [45], mutation [46], local search [15], and differential evolution [47, 48], were adopted in PSO as well.

An intelligent integration of different learning strategy in to the swarm evolutionary process is a promising direction for designing efficient PSO variants. Usually one prepares a collection of learning strategies, which possess different capabilities, such as exploitation, exploration, and jumping out from local optimum, and then, through a sophisticated adaptation mechanism, enables each particle to automatically choose learning strategies to determine the next move. Many state-of-the-art PSO variants have been developed following this development strategy. Liang et al. [35] proposed a comprehensive learning particle swarm optimizer (CLPSO), which uses a novel learning strategy whereby all other particles' historical best information is used to update a particle's velocity. Wang et al. [24] proposed a self-adaptive learning based PSO (SLPSO). SLPSO adopts four adaptive learning mechanisms, which are automatically chosen by particles based on each strategy's past performance. In [4], Zhan et al. proposed an orthogonal learning (OL) strategy for PSO to discover more useful information that lies in two particles' experiences via orthogonal experimental design. Experimental results demonstrated that OLPSO significantly improves the performance of PSO, offering faster convergence, higher solution quality, and stronger robustness. Hu et al. [18] proposed a PSO variant by intelligently combining a nonuniform mutation-based method and an adaptive subgradient method. A Cauchy mutation operator was further utilized to prevent premature convergence. Wang et al. [49] presented an enhanced PSO variant called GOBL, which employed generalized opposition-based learning (GOBL) and Cauchy mutation to overcome the deficiency of premature. Li et al. [19] presented a self-learning particle swarm optimizer, in which each particle has four strategies to cope with different situations in the search space. An adaptive cooperation mechanism was implemented at the individual level, which enables a particle to choose the rational strategy according to its own local fitness landscape.

## 3. The Inner Variable Learning Strategy

In this section, we introduce the knowledge employed in the inner variable learning (IVL) strategy and discuss the detailed implementation of the IVL strategy.

### 3.1. Knowledge Employed in the Inner Variable Learning Strategy

As mentioned before, adaptive learning is an important concept in designing evolutionary algorithms. In the basic PSO, each particle flies through the search space aiming to obtain a satisfactory solution, with its velocity and position being dynamically updated referring to its flying experience and its companions' experience. Two typical learning strategies are included in basic PSO: the first one is the self-learning strategy, which enables each particle to consider its past velocity and personal local best position when determining the next search direction and speed; the second one is the companion learning strategy, by which each particle takes into account the flying experience of its companions (such as learning from the global best position or all its neighbors' positions) in its space search process. It can be found from the review in Section 2 that current learning strategies (or cooperation and interaction) of PSO mainly happen at the swarm or particle level. However, the learning strategy at the variable level is rarely studied. We think that variable level based learning mechanisms would be more effective, since different variables of a particle are evolved independently. In addition, it is also meaningful to extract useful knowledge from the optimization problem to provide more exact and effective guidance for the search behavior of particles.

We find that, in many optimization functions, different variables come in the same form; that is, they are symmetric. Such functions usually combine the variables by using the operators of summation and product (“∑” and “∏”). For example, with regard to the Rosenbrock function: *f*(**x**) = ∑_*i*=1_^*D*^(100(*x*_*i*_^2^ − *x*_*i*+1_)^2^ + (*x*_*i*_ − 1)^2^), this function is multimodal and nonseparable and exhibits a very narrow valley moving from local optimum to global optimum [50]. Note that different variables in the Rosenbrock function are symmetric, since we can exchange the positions of any two variables *x*_*i*_ and *x*_*j*_ without affecting the function. Then, in the optimal solution, any two variables *x*_*i*_ and *x*_*j*_ are supposed to satisfy the relationship *x*_*i*_ = *x*_*j*_. More generally, let us consider an optimization function *f*(**a** **x** − **b**), where **a** = [*a*_1_, *a*_2_,…, *a*_*D*_], **x** = [*x*_1_, *x*_2_,…, *x*_*D*_], and **b** = [*b*_1_, *b*_2_,…, *b*_*D*_]. Let us formally define the concept of variable symmetry.

*Variable Symmetry.* two variables are symmetric if they can exchange their positions in the function through some linear transformation without affecting this function.

For instance, with regard to the two variables *x*_*i*_ and *x*_*j*_ in a given function, if we exchange these two variables by letting *x*_*i*_ = (*a*_*j*_ *x*_*j*_ + *b*_*j*_ − *b*_*i*_)/*a*_*i*_ and *x*_*j*_ = (*a*_*i*_ *x*_*i*_ + *b*_*i*_ − *b*_*j*_)/*a*_*j*_ without changing the function, then we say variables *x*_*i*_ and *x*_*j*_ are symmetric. If *x*_*i*_ and *x*_*j*_ are symmetric, then, in the optimal solution, there should exist the relationship *a*_*i*_ *x*_*i*_ + *b*_*i*_ = *a*_*j*_ *x*_*j*_ + *b*_*j*_. As an example, let us take the Shift Rastrigin function. In Rastrigin *f*_2_(**x**) = ∑_*i*=1_^*n*^(*y*_*i*_^2^ − 10cos⁡(2*πy*_*i*_) + 10), *y*_*i*_ = *x*_*i*_ − *o*_*i*_, **o** is a shift vector. Consider the original Shift Rastrigin function with two variables:

(4) ((x1−o1)2−10cos⁡(2π(x1−o1))+10)   +((x2−o2)2−10cos⁡(2π(x2−o2))+10).

Then we let *x*_1_ = *x*_2_ − *o*_2_ + *o*_1_ and *x*_2_ = *x*_1_ − *o*_1_ + *o*_2_ and substitute them into the above form:

(5) ((x2−o2+o1−o1)2−10cos⁡(2π(x2−o2+o1−o1))+10)   +((x1−o1+o2−o2)2     −10cos⁡(2π(x1−o1+o2−o2))+10)

which gives rise to the expression

(6) ((x2−o2)2−10cos⁡(2π(x2−o2))+10) +((x1−o1)2−10cos⁡(2π(x1−o1))+10).

It becomes clear that (6) and (4) are equal. The same situation happens for any other two variables. Therefore, the Shift Rastrigin function is completely symmetric. Different variables in the optimal solution of the Shift Rastrigin function have to satisfy *x*_*i*_ − *o*_*i*_ = *x*_*j*_ − *o*_*j*_.

It should be noted that the relation of variable symmetry is reflexive and transitive. We can determine the property of variable symmetry of the optimized function by exchanging positions of any two variables and checking the properties of reflexivity and transitivity. Sometimes, intuitive hints are also helpful.

Having noted the knowledge that symmetric variables of the optimal solution of a function should satisfy a certain quantitative relation, we can develop an inner variable learning (IVL) strategy in which different variables in the same function can realize learning from each other during the problem solving process. The idea of this learning strategy is simple and straightforward. Namely, in the course of learning, we check the variables of a particle and determine which variable's value is the best exemplar of other variables to optimize the function to the highest extent.

### 3.2. Implementation of the Inner Variable Learning Strategy

The previous learning strategies, such as the self-learning strategy and companion learning strategy, enable particles to learn their past flying experience or their companions' past flying experience, which are at the swarm or particle level. In comparison, the new learning strategy to be presented here is realized at the variable level and thus referred to as inner variable learning (IVL) strategy. The IVL strategy enables a particle to inspect the relation among its variables of the position, determine the exemplar variable for other variables, and then make each variable learn from the exemplar variable by modifying the values of other variables in terms of their quantitative relation with the exemplar variable and the value of the exemplar variable. This strategy will lead particles to fly to a better position quickly. Note that this learning strategy has originated from the knowledge of variable symmetry of optimized functions, such that it can be applied to any function involving symmetric variables.

If we execute the IVL strategy on each particle at every generation of PSO, it could be a little time consuming since every time we need to evaluate the effectiveness of each variable and select out an exemplar variable. In addition, performing the IVL strategy too frequently may cause PSO to suffer from premature convergence and make it get trapped in a local optimum at the early stage. That is because once a particle executes the IVL strategy, all its variables will be directly modified according to the value of the exemplar variable. In this study, the particle executes the IVL strategy immediately after it visits the personal best position or jumps out from a local optimum. This is because under these two occasions, the particle may have potential high-quality exemplar variable.

At each evolutionary generation, once the particle *i* determines its personal best position or executes the trap jumping operation, it will execute the inner learning strategy accordingly. Assume that the current personal best position and the corresponding velocity of particle *i* are **x**_**i**_ = [*x*_*i*1_, *x*_*i*2_,…, *x*_*iD*_] and **v**_**i**_ = [*v*_*i*1_, *v*_*i*2_,…, *v*_*iD*_]. We test the effectiveness of every variable *x*_*id*_ in the optimization function and take the best variable as the exemplar variable. That is to say, the exemplar variable is determined by first trying to let every variable be the exemplar variable and then ascertaining the best one as the ultimate exemplar variable. To obtain the effectiveness of variable *x*_*id*_, we just let *x*_*id*_ be the temporal exemplar variable, modify the value of any other variable *x*_*ik*_ according to *x*_*ik*_ = (*a*_*d*_ *x*_*id*_ + *b*_*d*_ − *b*_*k*_)/*a*_*k*_, and then calculate the function fitness. The temporary exemplar variable resulting in the best function fitness will be the ultimate exemplar variable. Assume that the ultimate exemplar variable is denoted by *d*. The procedure of the IVL strategy of particle *i* is described in Algorithm 1. Note that when a particle executes the IVL strategy once, it performs *D* evaluations of the fitness function.

## 4. The Trap Detection and Jumping out Techniques

The PSO algorithm is easy to implement and has been empirically shown to perform well on numerous optimization problems. However, it may easily get trapped in a local optimum when solving complex multimodal problems [35], such that effective mechanisms for particle detecting and jumping out of trap state become necessary.

Many researchers noted that it is helpful to improve the performance of PSO by intelligently tuning the particle's behaviors according to the current evolutionary states, which is usually evaluated by the statistic information of the swarm's distribution. For instance, Zhan et al. [17] determined the evolutionary states (i.e., exploration, exploitation, convergence, and jumping out) with the statistic of the position distribution information of the population, which was used for guiding the automatic parameter adjustment. The distribution information was obtained by calculating the mean distance of each particle to all the other particles. Chen et al. [51] also used the distance between particles to evaluate the diversity of PSO in the evolutionary process. They incorporated diversity into the objective function to optimize the optimization problem as well as guarantee the diversity of the overall swarm. It can be seen that the above methods are established at the swarm level, which means that the authors checked the diversity (or distribution information) of the overall swarm each time and adjusted the behaviors of particles accordingly. This may be not good for the adaptation and flexibility of a single particle, though. Although sometimes the diversity of the whole swarm is satisfactory according to certain criteria, some of the particles may actually have been trapped in optima.

To enable a particle to react to its solution space search situation more efficiently, we will check the diversity of particle *i* when *g*Best_*i*_ has not been improved continuously for *M* generations. In addition, let *m*_*i*_ denote the number of stagnation generation of particle *i*. At each generation, if the *g*Best_*i*_ has not improved, *m*_*i*_ is increased by 1; otherwise, *m*_*i*_ will be set to 1. Two criteria are considered to determine whether the particle is trapped in a local optimum.

Now let us take particle *i* as an example. The first criterion concerns the difference between the function fitness of the previous position **x**_*i*_′′ and that of the current position **x**_**i**_ of particle *i*. Let *δ* denote a threshold value of the difference of function fitness. In our study, we set *δ* = *α* · *t*/maxEF, where maxEF denotes the maximum number of function fitness evaluations, *t* denotes the current consumed number of function fitness evaluations, and *α* is scale coefficient. *δ* changes adaptively with the evolution of particles. If the function fitness difference is lower than *δ*, the particle may be considered to be trapped in a local optimum:

(7) $$\begin{array}{c} \left|f\left(x_{i}^{\prime \prime }\right)-f\left(x_{i}\right)\right|<\delta . \end{array}$$

The second criterion is as follows: if the distance between two consecutive positions of particle *i* is smaller than a predefined threshold value *ξ*, particle *i* may have been trapped:

(8) $$\begin{array}{c} ||x_{i}^{\prime \prime }-x_{i}||=\frac{\sqrt{\sum_{d}^{D}{\left(x_{id}^{\prime \prime }-x_{id}\right)}^{2}}}{D<\xi }. \end{array}$$

We set $\xi =\sqrt{D\cdot \beta^{2}}$, where *D* is the number of variables and *β* is the scale coefficient.

However, none of above two criterions can judge the trap state independently. That is because, on the one hand, as for the first criterion, a particle may have very close function fitness at two distant positions (meaning the particle is not trapped). On the other hand, the optimization function may be very sensitive to the landscape, so small deviation of the position of a particle may result in significant difference (similarly, meaning the particle is not trapped) of the function fitness. Therefore, the two criterions should be taken into account simultaneously. And if both of the above criterions are met, particle *i* is safely considered to be trapped in a local optimum.

Once we detect that a particle is trapped, a mutation operator will be employed to help particles to escape from the local optimum. Mutation is an indispensable operator in Genetic Algorithm. Due to the effectiveness of mutation operator in enhancing the diversity of population-based algorithms, it is also popularly adopted in many PSO variants. Generally, Cauchy mutation [18, 51] and Gaussian mutation [17, 49] methods are mostly used. Andrews [46] utilized a PSO algorithm incorporating different mutation operators to cope with both mathematical and constrained optimization problems. His results showed that the addition of a mutation operator to PSO could enhance optimization performance and insight was gained into how to design mutation operators dependent on the nature of the problem being optimized. The Gaussian mutation operator is utilized in the discussed PSO variant:

(9) $$\begin{array}{c} x_{id}^{\prime }=x_{id}+\omega \cdot \left(x_{\max,d}-x_{\min,d}\right)\cdot \lambda \cdot \text{Gaussian}\left(0,1\right), \end{array}$$

where *x*_max⁡,*d*_ and *x*_min⁡,*d*_ stand for the upper and low bound of the *d*th variable of the optimization problem and *ω* and *λ* are coefficients controlling the mutation scale:

(10) $$\begin{array}{c} \lambda =1-\frac{\sigma \cdot t}{\text{maxEF}}. \end{array}$$

Like the inertia weight parameter, mutation scale parameter *λ* linearly decreases with the evolutionary process, that is, starts declining from 1.0 to (1 − *σ*) gradually. *σ* (0 < *σ* < 1) reflects the decreased speed. The linear decrease of the mutation scale parameter enables the PSO to exhibit higher exploration capability at the early stage of the evolution and strong exploitation ability at the later evolutionary stage.

In addition, if *g*Best_*i*_ has not been improved for each *N* successive generation, particle *i* will perform the mutation operator. The PSO variant integrated with the IVL strategy, trap detection and jumping out strategy, and the basic PSO is shown as Algorithm 2.

## 5. Experimental Tests

### 5.1. Experimental Setting

In order to evaluate the performance of PSO-IVL, we compare it with some other state-of-the-art PSO alternatives. The parameters of the algorithms selected for comparison are summarized in Table 1. For comparison, the experimental settings of benchmark PSO variants are similar to that of [24]; that is, the population size of particles is 50 and the number of variables (dimensions) of each test function is set to 30, which is a typical setting encountered in the literature. Choosing proper parameters for an evolutionary algorithm is always time consuming since parameters are always related. According to analysis of extensive experimental work, the parameters of the PSO-IVL algorithm are specified as follows: *ps* = 20, maxEF = 300,000, *c*_1_ = *c*_2_ = 2.0, *α* = 0.1, *δ* = 0.1 · *t*/maxEF, *β* = 0.1, $\xi =\sqrt{0.01D}$, *ω* = 0.05, *σ* = 0.9, *λ* = 1 − (0.9 · *t*/maxEF), *M* = 50, and *N* = 200.

To realize a comprehensive analysis of the PSO-IVL and other PSO variants listed above, we conducted a series of experiments by employing 18 classical numerical optimization problems with different characteristics, including unimodality, multimodality, rotation, ill-conditionality, misscale, and noise. The optimization functions used in the experiments are listed below; **M** is the orthogonal matrix and **o** is the shifted vector:

 Sphere: *f*_1_(*x*) = ∑_*i*=1_^*n*^ *y*_*i*_^2^, **y** = **x** − **o**,
 Rastrigin: *f*_2_(**x**) = ∑_*i*=1_^*n*^(*y*_*i*_^2^ − 10cos⁡(2*πy*_*i*_) + 10), **y** = **x** − **o**,
 Rosenbrock: *f*_3_(**x**) = ∑_*i*=1_^*n*^(100(*y*_*i*_^2^ − *y*_*i*+1_)^2^ + (*y*_*i*_ − 1)^2^), **y** = **x** − **o**,
 Griewank: $f_{4}(z)=(1/4000)\sum_{i=1}^{n}y_{i}^{2}+1-\prod_{i=1}^{n}\cos(y_{i}/\sqrt{i})$, **y** = **x** − **o**,
 Ackley: $f_{5}(x)=-20\cdot \exp(-0.2\sqrt{\left(1/n\right)\sum_{i=1}^{n}y_{i}^{2}})+20-\exp((1/n)\sum_{i=1}^{n}\cos(2\pi y_{i}))+e$, **y** = **x** − **o**,
 Schwefel 1.2: *f*_6_(*x*) = ∑_*i*=1_^*n*^(∑_*j*=1_^*i*^ *y*_*j*_^2^), **y** = **x** − **o**,
 Scaled Rosenbrock 100: *f*_7_(*x*) = ∑_*i*=1_^*n*^(100((*a*_*i*_ *y*_*i*_)^2^ − (*a*_*i*+1_ *y*_*i*+1_))^2^ + (*a*_*i*_ *y*_*i*_ − 1)^2^), *a*_*i*_ = 100^(*i* − 1)/(*n*−1)^, **y** = **x** − **o**,
 Scaled Rastrigin 10: *f*_8_(**x**) = ∑_*i*=1_^*n*^((*a*_*i*_ *y*_*i*_)^2^ − 10cos⁡(2*π*(*a*_*i*_ *y*_*i*_)) + 10), *a*_*i*_ = 10^(*i* − 1)/(*n*−1)^, **y** = **x** − **o**,
 Noise Schwefel 1.2: *f*_9_(*x*) = ∑_*i*=1_^*n*^(∑_*j*=1_^*i*^ *y*_*j*_^2^) · (1 + 0.4 | *N*(0,1)|), **y** = **x** − **o**,
 Rotated Sphere: *f*_10_(*x*) = ∑_*i*=1_^*n*^ *z*_*i*_^2^, **z** = **M** · (**x** − **o**),
 Rotated Schwefel 2.21: *f*_11_(*x*) = max⁡|*z*_*i*_|, **z** = **M** · (**x** − **o**),
 Rotated Ellipse: *f*_12_(*x*) = ∑_*i*=1_^*n*^(20^(*i* − 1)/(*n*−1)^ *z*_*i*_)^2^, **z** = **M** · **x**,
 Rotated Rosenbrock: *f*_13_(**x**) = ∑_*i*=1_^*n*^(100(*z*_*i*_^2^ − *x*_*i*+1_)^2^ + (*z*_*i*_ − 1)^2^), **z** = **M** · (**x** − **o**),
 Rotated Ackley: $f_{14}(x)=-20\cdot \exp(-0.2\sqrt{(1/n)\sum_{i=1}^{n}z_{i}^{2}})+20-\exp((1/n)\sum_{i=1}^{n}\cos(2\pi z_{i}))+e$, **z** = **M** · (**x** − **o**),
 Rotated Griewank: $f_{15}(z)=(1/4000)\sum_{i=1}^{n}z_{i}^{2}+1-\prod_{i=1}^{n}\cos(z_{i}/\sqrt{i})$, **z** = **M** · (**x** − **o**),
 Rotated Rastrigin: *f*_16_(**x**) = ∑_*i*=1_^*n*^(*z*_*i*_^2^ − 10cos⁡(2*πz*_*i*_) + 10), **z** = **M** · (**x** − **o**),
 Noise Rotated Schwefel 1.2: *f*_17_(*x*) = ∑_*i*=1_^*n*^(∑_*j*=1_^*i*^ *z*_*j*_^2^) · (1 + 0.4 | *N*(0,1)|), **z** = **M** · (**x** − **o**),
 Noise Rotated Quadric: *f*_18_(*x*) = ∑_*i*=1_^*n*^ *iz*_*i*_^4^ + random[0,1), **z** = **M** · (**x** − **o**).

### 5.2. Comparative Analysis

The simulation results for each optimized function produced by PSO-w, PSO-cf, PSO-cf-local, FIPS-PSO, CPSO-H, CLPSO, and SLPSO are reported from [24]. Each optimization function is run by each PSO variant 30 times. The computational results are listed in Table 2 including the average value of the results along with their standard deviation. Suc denotes the number of successful runs. According to [24], a run is considered to be successful (i.e., has obtained a satisfactory solution) if a solution is obtained whose fitness value is not worse than (*fit*(x*)+(1.0*E* − 5)), where x* is the theoretical global optimal solution. FEs denotes the average number of function evaluations required to find the satisfactory solution when all 30 runs are successful.

From the computational results given in Table 2, we can conclude that PSO-IVL produced the best result for every test function. However, for Sphere function *f*_1_(*x*), Ackley function *f*_5_(*x*), Rotated Sphere function *f*_10_(*x*), and Rotated Ackley function *f*_14_(*x*), although PSO-IVL can find the optimal solution, its efficiency is not the highest. Moreover, PSO-IVL can find the optimal solution for all optimization functions only except Scaled Rosenbrock 100 *f*_7_(*x*), Rotated Rosenbrock *f*_13_(**x**), and Noise Quadric *f*_18_(*x*). Especially for some noisy and rotated functions, such as Noise Schwefel 1.2 *f*_9_(*x*), Rotated Ellipse *f*_12_(*x*), Rotated Rastrigin *f*_16_(**x**), and Noisy Rotated Schwefel 1.2 *f*_17_(*x*), other peer PSO variants cannot effectively acquire satisfactory solutions; however, PSO-IVL is successful in these cases. As a result, based on the reported results, Table 2, we conclude that the performance of PSO-IVL reported on the test functions is fairly competitive compared to other PSO variants.

### 5.3. Convergence Analysis of PSO-IVL

**Figure 1:**
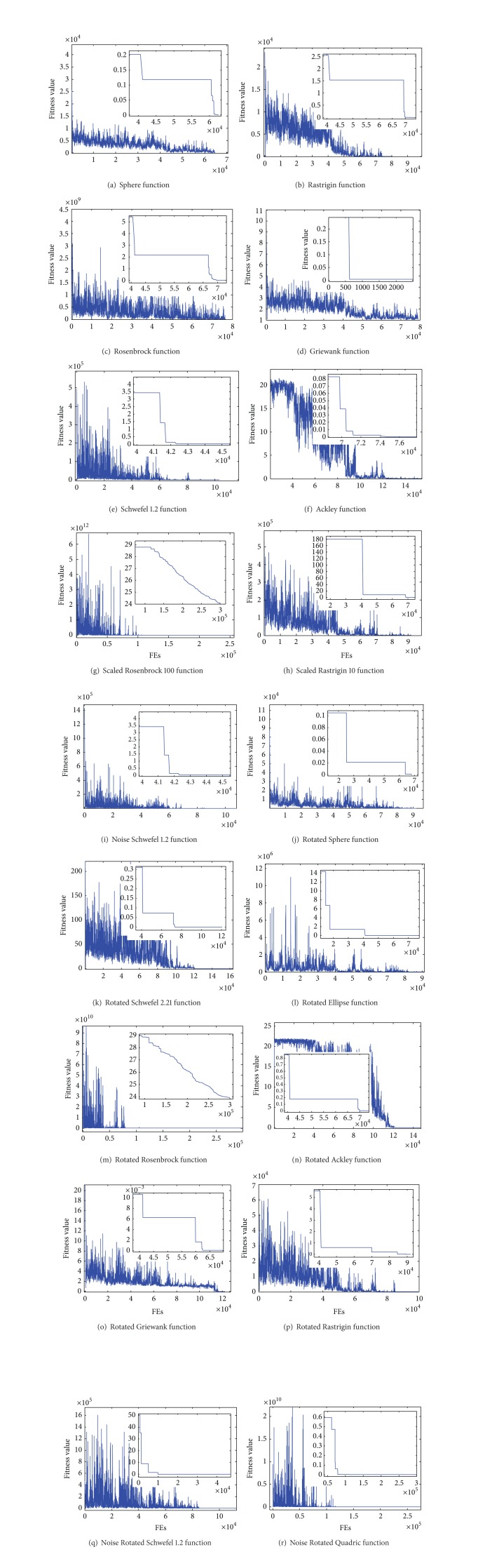
Evolutionary process of a particle and the global best solution with regard to each test function.

To provide an intuitive illustration of the optimization behavior of PSO-IVL, in Figure 1, we display the evolutionary process of a particle and the global-best-so-far solution when PSO-IVL is utilized to solve each optimization function. It should be noted that in the basic PSO and many typical PSO variants, for a particle, the number of generations and the number of fitness evaluations are usually equal. However, the situation is different in our study. At any generation, if the particle does not perform the IVL strategy, a single fitness evaluation is required; thus in this case one generation is corresponding to one fitness evaluation. In comparison, if the particle executes the IVL strategy at a given generation, then there are *D* (*D* is the number of variables associated with the functions considered) numbers of fitness evaluation operation to complete. As a result, in this situation, one generation is related to *D* of fitness evaluations. The evolutionary process of other PSO alternatives can be found in [24].

The larger figure shows how the fitness of the position visited by a particle in PSO-IVL changes with the increase of the number of function fitness evaluations while the smaller figure visualizes how the fitness of the global-best-so-far solution evolves. Since the global-best-so-far solution converges to a good value very fast, it would be unclear to see its overall evolution. We enlarge and display the evolutionary process of global-best-so-far solution at some stages.

Two observations are worth making here. First, the fitness of the particle fluctuates quite substantially at the early stage of PSO-IVL but gradually diminishes and finally converges to the optimal solution. This is because of the trap detection and jumping out strategy adopted in PSO-IVL. During the evolutionary process, if the particle is detected to be trapped in a local optimum, the particle performs Gaussian mutation, which adaptively enables the particle to randomly move to a new position. The adaptive Gaussian mutation operator makes the particle fluctuate to a large extent in the early stage of optimization. In addition, the learning and interaction strategies realized within the swarm enable the particle to always converge to a good solution.

Second, the particle exhibits a certain probability to determine high-quality solutions at the early stage. The reason is that PSO-IVL employs the IVL strategy, which enables the particle to learn among different variables. As a result, just a good value of a variable can quickly lead the particle to reach a position with good fitness.

The smaller figures indicate that PSO-IVL can converge to a high-quality solution fast on each test function and finally find the optimal solutions for most functions. This can be explained by the fact that the IVL strategy indeed enables the particle to find high-quality position at high speed and high probability; meanwhile, the trap detection and jumping out strategy can help particles escape from local optima. As a conclusion, the combination of the IVL strategy, the trap detection and jumping out strategy, and the basic PSO forms an efficient optimization environment.

### 5.4. Analysis of the Impact of the Inner Variable Learning Strategy

As we know, some optimization functions may be partial symmetric. In this case, when we use PSO-IVL to carry out optimization, only a portion of their variables can be utilized to realize the IVL strategy. Therefore, it is important to investigate the impact of the number of variables being involved in the IVL strategy. For convenient comparison, we selected six complex optimization functions (where it is hard to obtain an optimal or near optimal solution for these functions without using the IVL strategy) and solved them by using PSO-IVL with a different number of variables involved in the IVL strategy. This means that, even though in these functions all variables are symmetric, each time we only set a certain number of variables to run the IVL strategy. The obtained results are listed in Table 3, where *n* stands for the number of variables executed by the IVL strategy. We set *n* to 0, 5, 10, 15, 25, and 30, respectively. When *n* is equal to 0, this means that in fact the IVL strategy is not invoked. When *n* equals 30, all variables are viewed as symmetric and adopted to execute the IVL strategy.

From the results displayed in Table 3, we can find that, for every selected optimization function, the solution obtained by PSO-IVL is getting better with the increase of the values of *n*. Therefore, we can come to some conclusions. (1) The effectiveness of the IVL strategy is significant. (2) More variables in an optimization function being utilized the IVL strategy (i.e., more variables are symmetric) will lead to much better solutions. (3) Even if there are only less symmetric variables in an optimization function, the employment of the IVL strategy on these variables has potential to improve the optimization process.

## 6. Conclusions

In this work, we have introduced a new knowledge-driven PSO variant (PSO-IVL), which integrates the generic PSO, a novel inner variable learning (IVL) strategy, and a novel trap detection and jumping out strategy. The IVL strategy is based on the knowledge that the values of symmetric variables in an optimization function will satisfy certain relations in the optimal solution. The trap detection and jumping out strategy is established at the level of individual particles rather than the swarm level, which improves the flexibility and adaptability of particles and helps particles escape from local optima. Experimental simulations completed for some classical optimization functions demonstrate the competitive performance of PSO-IVL, which is superior to all the selected state-of-the-art peer PSO variants.

Although we choose completely symmetric functions in which all variables are symmetric to test our algorithm's performance, the proposed algorithm can also be applied to partial symmetric functions (in which only some variables are symmetric). In this case, we just need to let the IVL strategy be performed on the symmetric variables. Moreover, the IVL strategy can be integrated into existing PSO alternatives.

PSO-IVL will be effective in optimization functions possessing symmetric variables. However, it is meaningful for three reasons. Firstly, it can obtain good solutions (usually the optimal solutions) for many benchmark functions. Secondly and more importantly, the efficiency of PSO-IVL indicates that the combination of the problem-oriented knowledge and PSO would be a promising direction for applying PSO to optimization problems. Thirdly, since symmetry is a general phenomenon existing in nature and engineering, it could be beneficial to check the variable symmetry when we try to use PSO or other evolutionary algorithms to solve a new complex optimization problem.

The future research can be carried out in three directions. One can look at discovering and formalizing domain knowledge (e.g., generic quantitative relations among different variables) existing in optimization problems and integrate it into the design of more advanced PSO schemes. The second one is to attempt to apply PSO-IVL to some real-life optimization problems. The third direction could be to formulate a general framework for guiding knowledge discovery in optimization problems and its integration into evolutionary algorithms.

## Figures and Tables

**Algorithm 1:**
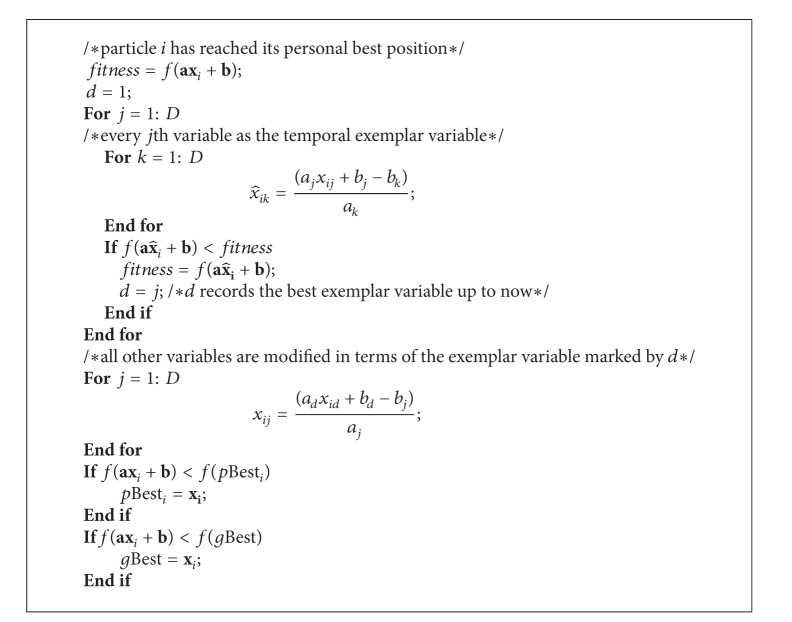
InnerVariableLearning(*i*).

**Algorithm 2:**
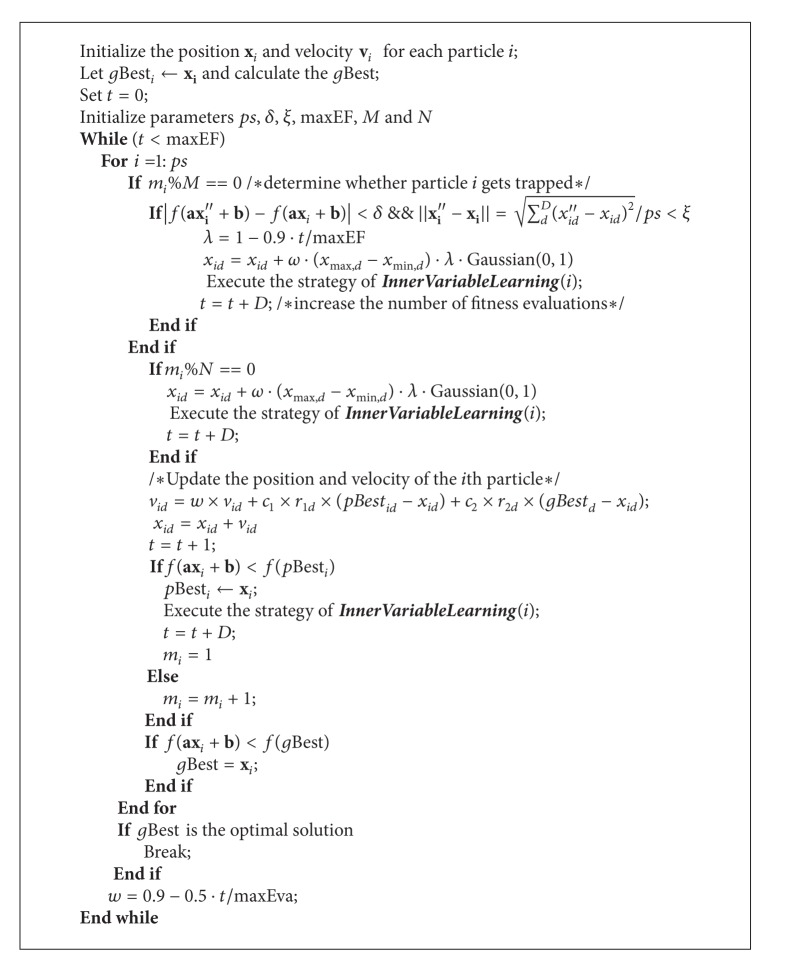
Procedure of PSO-IVL.

**Table 1:** PSO variants used in comparative studies.

PSO variants	Parameters setting
PSO-w: PSO with inertia weight [31]	$w=0.9-\frac{0.5\cdot g}{\text{maxGen}}$, *f*_1_ = *f*_2_ = 1.49
PSO-cf: PSO with constriction factor [32]	*w* = 0.729, *c*_1_ = *c*_2_ = 1.49445
PSO-cf-local: local version of PSO with constriction factor [16]	*w* = 0.729, *c*_1_ = *c*_2_ = 1.49445
FIPS-PSO: fully informed PSO [33]	*w* = 0.729, *c*_1_ = *c*_2_ = 2.0
CPSO-H: cooperative based PSO [34]	$w=0.9-\frac{0.5\cdot g}{\text{maxGen}}$, *c*_1_ = *c*_2_ = 2.0
CLPSO: comprehensive learning PSO [35]	$w=0.9-\frac{0.5\cdot g}{\text{maxGen}}$, *c*_1_ = *c*_2_ = 1.49445
SLPSO: self-adaptive learning based Particle Swarm Optimization [24]	*pr* *oS* *TR*_*i*_ = 0.25, *Gs* = 10, *w*_*i*_ = log⁡(*ps* − *i* + 1)/(log⁡(1) + ⋯+log⁡(*ps*))

**Table 2:** Optimization results obtained for the test functions; the best results are shown in boldface.

Functions Algorithm	Mean	StdDev	Suc	FEs	Mean	StdDev	Suc	FEs	Mean	StdDev	Suc	FEs
	Sphere				Rastrigin				Rosenbrock			
PSO-w	0.00*E* + 00	0.00*E* + 00	30	193,017	1.75*E* + 01	5.97*E* + 00	0		6.46*E* + 01	8.01*E* + 01	0	
PSO-cf	**0.00*E* + 00**	**0.00*E* + 00**	**30**	**20,333**	6.45*E* + 01	2.05*E* + 01	0		8.88*E* + 00	1.46*E* + 01	0	
PSO-cf-local	0.00*E* + 00	0.00*E* + 00	30	40,295	4.10*E* + 01	1.13*E* + 01	0		2.98*E* + 01	4.07*E* + 01	0	
FIPS-PSO	0.00*E* + 00	0.00*E* + 00	30	146,179	6.39*E* + 01	1.12*E* + 01	0		2.52*E* + 01	9.08*E* − 01	0	
CPSO-H	0.00*E* + 00	1.49*E* − 08	30	149,044	3.32*E* − 02	1.82*E* − 01	29		2.77*E* + 01	2.86*E* + 01	0	
CLPSO	0.00*E* + 00	0.00*E* + 00	30	122,161	0.00*E* + 00	0.00*E* + 00	30	195,815	4.95*E* + 00	3.79*E* + 00	0	
SLPSO	0.00*E* + 00	0.00*E* + 00	30	43,980	0.00*E* + 00	0.00*E* + 00	30	196,749	2.66*E* − 01	1.01*E* + 00	8	
PSO-IVL	0.00*E* + 00	0.00*E* + 00	30	81,950	**0.00*E* + 00**	**0.00*E* + 00**	**30**	**99,430**	**0.00*E* + 00**	**0.00*E* + 00**	**30**	**136,250**

Algorithms	Griewank				Schwefel 1.2				Ackley			

PSO-w	8.73*E* − 02	1.18*E* − 01	2		0.00*E* + 00	1.11*E* + 00	0		2.18*E* + 05	4.83*E* − 14	30	211,209
PSO-cf	1.71*E* − 02	1.78*E* − 02	8		1.29*E* − 12	3.88*E* − 11	30	151,095	8.51*E* − 01	1.01*E* + 00	15	
PSO-cf-local	5.34*E* − 03	7.46*E* − 03	17		1.53*E* − 04	1.68*E* − 04	1		0.00*E* + 00	0.00*E* + 00	30	56,976
FIPS-PSO	2.72*E* − 07	1.18*E* − 06	30	183,581	2.08*E* + 02	8.98*E* + 01	0		1.39*E* − 08	2.98*E* − 09	30	
CPSO-H	1.20*E* − 01	2.18*E* − 01	4		2.79*E* + 03	5.98*E* + 03			2.44*E* − 05	1.35*E* − 05	1	
CLPSO	0.00*E* + 00	0.00*E* + 00	30	151,708	1.16*E* + 03	2.44*E* + 02	0		7.77*E* − 13	1.49*E* − 13	30	166,425
SLPSO	1.81*E* − 03	4.79*E* − 03	26		7.69*E* − 13	4.27*E* − 13	30	149,872	**0.00*E* + 00**	**0.00*E* + 00**	**30**	**51,585**
PSO-IVL	**0.00*E* + 00**	**0.00*E* + 00**	**30**	**81,960**	**0.00*E* + 00**	**0.00*E* + 00**	**30**	**92,600**	0.00*E* + 00	0.00*E* + 00	30	83,330

Algorithms	Scaled Rosenbrock 100				Scaled Rastrigin 10				Noise Schwefel 1.2			

PSO-w	2.18*E* + 05	8.24*E* + 05	0		2.18*E* + 05	6.59*E* + 00	0		4.68*E* + 02	3.14*E* + 02	0	
PSO-cf	2.57*E* + 04	4.91*E* + 04	0		2.57*E* + 04	2.85*E* + 01	0		1.99*E* + 02	2.89*E* + 02	0	
PSO-cf-local	9.11*E* + 04	3.72*E* + 05	0		2.57*E* + 04	1.35*E* + 01	0		5.71*E* + 02	4.59*E* + 02	0	
FIPS-PSO	7.37*E* + 04	3.14*E* + 05	0		7.37*E* + 04	9.23*E* + 00	0		1.52*E* + 03	5.44*E* + 02	0	
CPSO-H	3.71*E* + 06	4.38*E* + 06	0		1.23*E* + 07	1.86*E* − 07	0		2.44*E* + 04	8.49*E* + 03	0	
CLPSO	1.09*E* + 03	3.45*E* + 03	0		0.00*E* + 00	0.00*E* + 00	30	226,863	7.25*E* + 03	1.37*E* + 03	0	
SLPSO	7.88*E* + 02	2.56*E* + 03	0		0.00*E* + 00	0.00*E* + 00	30	234,253	2.32*E* − 02	8.92*E* − 02	0	
PSO-IVL	**2.32*E* + 01**	**2.54*E* − 03**	**0**		**0.00*E* + 00**	**0.00*E* + 00**	**30**	**83,**660	**0.00*E* + 00**	**0.00*E* + 00**	**30**	**97,530**

Algorithms	Rotated Sphere				Rotated Schwefel 2.21				Rotated Ellipse			

PSO-w	0.00*E* + 00	4.56*E* − 14	30	201,639	2.50*E* − 01	3.03*E* − 01	0		1.15*E* + 02	1.49*E* + 02	0	
PSO-cf	**0.00*E* + 00**	**0.00*E* + 00**	**30**	**24,010**	4.11*E* − 02	1.40*E* − 01	17		3.46*E* − 03	1.13*E* − 02	0	
PSO-cf-local	0.00*E* + 00	0.00*E* + 00	30	47,289	7.98*E* − 02	2.03*E* − 01	14		7.66*E* − 01	1.09*E* + 00	0	
FIPS-PSO	7.54*E* − 13	3.26*E* − 13	30		1.36*E* − 04	4.89*E* − 05	0		1.51*E* + 03	7.14*E* + 02	0	
CPSO-H	8.10*E* − 08	1.02*E* − 07	30		5.43*E* + 01	7.52 + 00	0		7.63*E* + 03	6.69*E* + 03	0	
CLPSO	4.21*E* − 10	6.18*E* − 10	30	190,125	9.71*E* − 01	2.38*E* − 01	0		4.07*E* + 03	9.37*E* + 02	0	
SLPSO	0.00*E* + 00	0.00*E* + 00	30	49,396	4.51*E* − 11	1.16*E* − 10	30	81,481	2.22*E* − 12	7.06*E* − 13	30	146,787
PSO-IVL	0.00*E* + 00	0.00*E* + 00	30	84,750	**0.00*E* + 00**	**0.00*E* + 00**	**30**	**129,880**	**0.00*E* + 00**	**0.00*E* + 00**	**30**	**97800**

Algorithms	Rotated Rosenbrock				Rotated Ackley				Rotated Griewank			

PSO-w	4.62*E* + 05	7.88*E* + 05	0		2.34*E* + 00	7.59*E* − 01	1		2.07*E* − 01	3.84*E* − 01	0	
PSO-cf	1.18*E* + 03	2.98*E* + 03	0		1.95*E* + 00	9.55*E* − 01	4		9.85*E* − 03	7.99*E* − 03	8	
PSO-cf-local	6.16*E* + 02	1.93*E* + 03	0		2.40*E* − 01	4.98*E* − 01	24		1.04*E* − 02	1.03*E* − 02	9	
FIPS-PSO	2.89*E* + 01	4.15*E* + 00	0		2.24*E* − 08	5.60*E* − 09	30	213,274	1.14*E* − 03	3.00*E* − 03	8	
CPSO-H	1.62*E* + 03	3.78*E* + 03	0		1.76*E* + 01	3.96*E* + 00	0		1.66*E* + 00	2.10*E* − 01	0	
CLPSO	2.56*E* + 02	3.04*E* + 02	0		1.16*E* − 02	9.10*E* − 03	0		3.36*E* − 02	2.00*E* − 02	0	
SLPSO	1.20*E* + 02	3.81*E* + 02	0		**0.00*E* + 00**	**0.00*E* + 00**	**30**	**52,575**	5.34*E* − 03	6.82*E* − 03	17	
PSO-IVL	**2.38*E* + 01**	**2.80*E* − 03**	**0**		0.00*E* + 00	0.00*E* + 00	30	95,230	**0.00*E* + 00**	**0.00*E* + 00**	**30**	**84910**

Algorithms	Rotated Rastrigin				Noise Rotated Schwefe1.2				Noise Quadric			

PSO-w	8.29*E* + 01	5.00*E* + 01	0		4.88*E* + 02	3.51*E* + 02	0		1.64*E* − 02	6.28*E* − 03	0	
PSO-cf	1.09*E* + 02	3.63*E* + 01	0		2.39*E* + 02	2.97*E* + 02	0		6.07*E* − 03	2.83*E* − 03	0	
PSO-cf-local	5.26*E* + 01	1.43*E* + 01	0		5.96*E* + 02	3.86*E* + 02	0		6.84*E* − 03	2.05*E* − 03	0	
FIPS-PSO	1.75*E* + 02	8.79*E* + 00	0		1.47*E* + 03	5.40*E* + 02	0		8.61*E* − 03	2.12*E* − 03	0	
CPSO-H	3.77*E* + 02	1.10*E* + 02	0		2.54*E* + 04	1.15*E* + 04	0		5.30*E* − 02	2.17*E* − 02	0	
CLPSO	1.03*E* + 02	1.26*E* + 01	0		6.96*E* + 03	1.49*E* + 03	0		1.86*E* − 02	7.92*E* − 03	0	
SLPSO	3.15*E* + 01	6.41*E* + 00	0		7.79*E* − 04	2.24*E* − 03	7		2.43*E* − 03	7.71*E* − 04	0	
PSO-IVL	**0.00*E* + 00**	**0.00*E* + 00**	**30**	**116,660**	**0.00*E* + 00**	**0.00*E* + 00**	**30**	**102,080**	**7.55*E* − 05**	**7.51*E* − 08**	**0**	

**Table 3:** Results of different numbers of variables being executed with the IVL strategy.

Functions	*n* = 0	*n* = 5	*n* = 10	*n* = 15	*n* = 20	*n* = 25	*n* = 30
Scaled Rosenbrock 100: *f*_7_	8.45*E* + 04	2.34*E* + 04	5.82*E* + 03	3.65*E* + 03	1.48*E* + 02	7.55*E* + 01	2.32*E* + 01
Noise Schwefel 1.2: *f*_9_	9.82*E* + 02	6.37*E* + 01	1.46*E* + 00	8.17*E* − 04	3.25*E* − 08	9.72*E* − 13	0.00*E* + 00
Rotated Schwefel 2.21: *f*_11_	6.63*E* − 01	1.16*E* − 01	3.61*E* − 02	8.28*E* − 04	2.52*E* − 08	1.07*E* − 14	0.00*E* + 00
Rotated Ellipse: *f*_12_	2.91*E* + 02	3.97*E* + 00	1.76*E* − 03	5.11*E* − 06	5.34*E* − 11	6.28*E* − 19	0.00*E* + 00
Rotated Rosenbrock: *f*_13_	1.82*E* + 04	8.82*E* + 03	2.57*E* + 03	9.07*E* + 02	2.93*E* + 02	5.64*E* + 01	2.87*E* + 01
Noise Rotated Schwefe1.2: *f*_17_	2.77*E* + 03	5.75*E* + 02	6.03*E* + 00	4.23*E* − 04	2.45*E* − 09	1.87*E* − 15	0.00*E* + 00

## References

[1] Kennedy J, Eberhart R Particle swarm optimization.

[2] Eberhart R, Kennedy J New optimizer using particle swarm theory.

[3] Eberhart R, Shi Y, Kennedy J (2001). *Swarm Intelligence*.

[4] Zhan Z-H, Zhang J, Li Y, Shi Y-H (2011). Orthogonal learning particle swarm optimization. *IEEE Transactions on Evolutionary Computation*.

[5] Eberhart R, Shi Y (1998). Comparison between genetic algorithms and particle swarm optimization. *Evolutionary Programming VII*.

[6] Shi Y, Eberhart R (1998). Parameter selection in particle swarm optimization. *Evolutionary Programming VII*.

[7] Chatterjee A, Siarry P (2006). Nonlinear inertia weight variation for dynamic adaptation in particle swarm optimization. *Computers & Operations Research*.

[8] Ratnaweera A, Halgamuge SK, Watson HC (2004). Self-organizing hierarchical particle swarm optimizer with time-varying acceleration coefficients. *IEEE Transactions on Evolutionary Computation*.

[9] Shi Y, Eberhart RC Fuzzy adaptive particle swarm optimization.

[10] Ismail A, Engelbrecht A (2012). The self-adaptive comprehensive learning particle swarm optimizer. *Swarm Intelligence*.

[11] Parsopoulos KE, Vrahatis MN (2007). Parameter selection and adaptation in unified particle swarm optimization. *Mathematical and Computer Modelling*.

[12] Kennedy J Small worlds and mega-minds: effects of neighborhood topology on particle swarm performance.

[13] Suganthan PN Particle swarm optimiser with neighbourhood operator.

[14] Hu X, Eberhart R Multiobjective optimization using dynamic neighborhood particle swarm optimization.

[15] Liang JJ, Suganthan PN Dynamic multi-swarm particle swarm optimizer.

[16] Kennedy J, Mendes R Population structure and particle swarm performance.

[17] Zhan Z-H, Zhang J, Li Y, Chung HS-H (2009). Adaptive particle swarm optimization. *IEEE Transactions on Systems, Man, and Cybernetics B*.

[18] Hu M, Wu T, Weir JD (2012). An intelligent augmentation of particle swarm optimization with multiple adaptive methods. *Information Sciences*.

[19] Li C, Yang S, Nguyen TT (2012). A self-learning particle swarm optimizer for global optimization problems. *IEEE Transactions on Systems, Man, and Cybernetics B*.

[20] Angeline P (1998). Evolutionary optimization versus particle swarm optimization: philosophy and performance differences. *Evolutionary Programming VII*.

[21] Wei C, He Z, Zhang Y, Pei W Swarm directions embedded in fast evolutionary programming.

[22] Yao X, Liu Y, Lin G (1999). Evolutionary programming made faster. *IEEE Transactions on Evolutionary Computation*.

[23] Poli R, Di Chio C, Langdon WB Exploring extended particle swarms: a genetic programming approach.

[24] Wang Y, Li B, Weise T, Wang J, Yuan B, Tian Q (2011). Self-adaptive learning based particle swarm optimization. *Information Sciences*.

[25] Xing L-N, Chen Y-W, Wang P, Zhao Q-S, Xiong J (2010). A knowledge-based ant colony optimization for flexible job shop scheduling problems. *Applied Soft Computing Journal*.

[26] Wu G, Liu J, Ma M, Qiu D (2013). A two-phase scheduling method with the consideration of task clustering for earth observing satellites. *Computers & Operations Research*.

[27] Wu G, Ma M, Zhu J, Qiu D (2012). Multi-satellite observation integrated scheduling method oriented to emergency tasks and common tasks. *Journal of Systems Engineering and Electronics*.

[28] Li H, Wu B (2013). Adaptive geo-information processing service evolution: reuse and local modification method. *ISPRS Journal of Photogrammetry and Remote Sensing*.

[29] Li H, Zhu Q, Yang X, Xu L (2012). Geo-information processing service composition for concurrent tasks: a QoS-aware game theory approach. *Computers & Geosciences*.

[30] Wu G, Pedrycz W, Li H, Qiu D, Ma M, Liu J (2013). Complexity reduction in the use of evolutionary algorithms to function optimization: a variable reduction strategy. *The Scientific World Journal*.

[31] Shi Y, Eberhart R Modified particle swarm optimizer.

[32] Clerc M, Kennedy J (2002). The particle swarm-explosion, stability, and convergence in a multidimensional complex space. *IEEE Transactions on Evolutionary Computation*.

[33] Mendes R, Kennedy J, Neves J (2004). The fully informed particle swarm: simpler, maybe better. *IEEE Transactions on Evolutionary Computation*.

[34] van den Bergh F, Engelbrecht AP (2004). A cooperative approach to participle swam optimization. *IEEE Transactions on Evolutionary Computation*.

[35] Liang JJ, Qin AK, Suganthan PN, Baskar S (2006). Comprehensive learning particle swarm optimizer for global optimization of multimodal functions. *IEEE Transactions on Evolutionary Computation*.

[36] Banks A, Vincent J, Anyakoha C (2007). A review of particle swarm optimization—part I: background and development. *Natural Computing*.

[37] Banks A, Vincent J, Anyakoha C (2008). A review of particle swarm optimization—part II: hybridisation, combinatorial, multicriteria and constrained optimization, and indicative applications. *Natural Computing*.

[38] Poli R, Kennedy J, Blackwell T (2007). Particle swarm optimization. *Swarm Intelligence*.

[39] del Valle Y, Venayagamoorthy GK, Mohagheghi S, Hernandez J-C, Harley RG (2008). Particle swarm optimization: basic concepts, variants and applications in power systems. *IEEE Transactions on Evolutionary Computation*.

[40] Rana S, Jasola S, Kumar R (2011). A review on particle swarm optimization algorithms and their applications to data clustering. *Artificial Intelligence Review*.

[41] Boeringer DW, Werner DH (2004). Particle swarm optimization versus genetic algorithms for phased array synthesis. *IEEE Transactions on Antennas and Propagation*.

[42] Shi Y, Eberhart RC Empirical study of particle swarm optimization.

[43] Kennedy J Particle swarm: social adaptation of knowledge.

[44] Juang C-F (2004). A hybrid of genetic algorithm and particle swarm optimization for recurrent network design. *IEEE Transactions on Systems, Man, and Cybernetics B*.

[45] Chen Y-P, Peng W-C, Jian M-C (2007). Particle swarm optimization with recombination and dynamic linkage discovery. *IEEE Transactions on Systems, Man, and Cybernetics B*.

[46] Andrews PS An investigation into mutation operators for particle swarm optimization.

[47] Zhang W-J, Xie X-F DEPSO: hybrid particle swarm with differential evolution operator.

[48] Omran MGH, Engelbrecht AP, Salman A Differential evolution based particle swarm optimization.

[49] Wang H, Wu Z, Rahnamayan S, Liu Y, Ventresca M (2011). Enhancing particle swarm optimization using generalized opposition-based learning. *Information Sciences*.

[50] Suganthan PN, Hansen N, Liang JJ (2005). *Problem Definitions and Evaluation Criteria for the CEC, 2005 Special Session on Real-Parameter Optimization*.

[51] Chen C-Y, Chang K-C, Ho S-H (2011). Improved framework for particle swarm optimization: swarm intelligence with diversity-guided random walking. *Expert Systems with Applications*.

